# Assessment of inflammatory markers in otosclerosis patients^☆^

**DOI:** 10.1016/j.bjorl.2018.12.014

**Published:** 2019-03-08

**Authors:** Cengiz Arli, Ihsan Gulmez, Elif Tuğba Saraç, Şemsettin Okuyucu

**Affiliations:** a Mustafa Kemal University, Medicine Faculty Antakya, Ear-Nose-Throat Department, Antakya, Turkey; b Hatay State Hospital, Ear-Nose-Throat Department, Antakya, Turkey; c Mustafa Kemal University, Audiology Department, Medicine Faculty Antakya, Antakya, Turkey; d Mustafa Kemal University, Medicine Faculty Antakya, Ear-Nose-Throat Department, Antakya, Turkey

**Keywords:** Otosclerosis, Neutrophil lymphocyte ratio, Platelet lymphocyte ratio, Mean platelet volume, Inflammatory markers, Otosclerose, Relação neutrófilos-linfócitos, Relação plaquetas-linfócitos, Volume plaquetário médio, Marcadores de inflamação

## Abstract

**Introduction:**

Otosclerosis is an idiopathic disease characterized by new bone formation in foci of the human otic capsule. It is more common in Caucasian populations; affecting females twice as often as males. Its etiopathogenesis has not yet been fully elucidated.

**Objective:**

The aim of this study was to investigate the relationship between otosclerosis and white blood cell and thrombocyte counts, mean platelet volume, neutrophil lymphocyte ratio, and the platelet lymphocyte ratio.

**Methods:**

This retrospective case-control study was conducted in the outpatient clinic Mustafa Kemal University, in the department of otolaryngology, between 2015 and 2018. A total of 30 patients with an established diagnosis of otosclerosis were compared to a control group of 30 healthy subjects, matched for age, gender and body mass index. The white blood cell, thrombocyte, mean platelet volume, neutrophil lymphocyte ratio and platelet lymphocyte ratio values were calculated for all participants.

**Results:**

There was no statistically significant difference between the groups with respect to age, gender, or body mass index, or for the mean neutrophil lymphocyte ratio, platelet lymphocyte ratio, white blood cell, or thrombocyte values (*p* > 0.05). A statistically significant difference was determined between the groups for the mean platelet volume values. The mean platelet volume values were lower in the otosclerotic patients (*p* = 0.047).

**Conclusion:**

These results show that neutrophil lymphocyte ratio, platelet lymphocyte ratio, white blood cell and thrombocytes should not be used to predict otosclerosis, but suggest that mean platelet volume may be a negative predictive marker.

## Resumo

**Introdução:**

A otosclerose é uma doença idiopática caracterizada por neoformação óssea em focos da cápsula ótica humana. É mais comum em populações caucasianas, afeta o sexo feminino numa taxa 2 vezes maior do que o masculino. A etiopatogenia ainda não foi totalmente elucidada.

**Objetivo:**

Investigar a relação entre otosclerose e taxa de glóbulos brancos, plaquetas, volume plaquetário médio, relação neutrófilos-linfócitos e relação plaquetas-linfócitos.

**Método:**

Estudo retrospectivo de caso-controle feito no ambulatório da Mustafa Kemal University, Departamento de Otorrinolaringologia, entre 2015 e 2018. Foram comparados 30 pacientes com diagnóstico estabelecido de otosclerose com um grupo controle de 30 indivíduos saudáveis, pareados por idade, sexo e índice de massa corpórea. Os valores de glóbulos brancos, plaquetas, volume plaquetário médio, relação neutrófilos-linfócitos e relação plaquetas/linfócitos foram calculados para todos os participantes.

**Resultados:**

Não houve diferença estatisticamente significante entre os grupos em relação a idade, sexo ou índice de massa corpórea, ou em relação aos valores médios de relação neutrófilos-linfócitos, relação plaquetas/linfócitos, valores de glóbulos brancos e plaquetas (*p* > 0,05). Uma diferença estatisticamente significante foi observada entre os grupos em termos de valores médios de volume plaquetário médio. Os valores de volume plaquetário médio foram menores nos pacientes com otosclerose (*p* = 0,047).

**Conclusão:**

A relação plaquetas/linfócitos, a relação plaquetas/linfócitos, os valores de glóbulos brancos e plaquetas não podem ser usados para predizer a otosclerose, mas sugerem que o volume plaquetário médio possa ser um marcador preditivo negativo.

## Introduction

Otosclerosis is an idiopathic disease characterized by formation of new bone in the otic capsule.^1^ The pathologic hallmark of the disease is an abnormal bony remodeling, that includes bone resorption, new bone deposition, and vascular proliferation in the temporal bone.^2^ Several etiological factors have been reported including genetic predisposition, impaired bone metabolism, inflammation and hormonal factors, and traumatic and autoimmune influences.^1^, ^3^ There are evidently genetic factors that can lead to this disease, but measles virus infection and autoimmunity may also play contributing roles.^4^ McKenna et al. found that the similarity between otosclerosis and Paget's disease due to a possible viral etiology. McKenna and Mills supported the hypothesis of a viral etiology for otosclerosis by means of ultrastructural and immunohistochemical evidence of measles-like structures and antigenicity in active otosclerotic lesions.^5^ The incidence of otosclerosis in the general population is 0.3–1%, with females affected at twice the rate of males. In patients with hearing loss it is seen at 5–9% and in those with conductive type hearing loss, at 18–22%.^1^

Although conductive type hearing loss is among the clinical findings, it can be associated with sensorioneural and mixed type hearing loss.^2^, ^3^ Abnormal bone formation and destruction are seen together in otosclerosis, and the involvement restricted to the otic capsule, differentiates it from diseases such as Paget's disease and osteogenesis imperfecta.^6^ In the histopathology, the most involved region is the *fissula antefenestram* in the anterior portion of the oval window. When the involved regions are examined, foci of abnormal bone resorption and deposition are observed. There are 3 phases in the development of ostesclerotic foci. These are the early osteospongiotic phase which is soft and vascular, the transition phase and the late phase which is avascular sclerotic.^6^

A cell-mediated immune response has an important role in the pathogenesis of several diseases. Independent prognostic factors are of great importance in the treatment and follow-up of these diseases. In this context, the neutrophil lymphocyte ratio (NLR), which is simple, cost-effective and has a high rate of accuracy, has been used in recent years as an important prognostic factor in inflammatory diseases in general, and prominently in cardiovascular system diseases and several types of cancer.^7^, ^8^

In ENT practice, a relationship has been found between NLR rates and various pathological states including vestibular neuritis, Bell's palsy, idiopathic sudden sensoneurial hearing loss, severe tinnitus and head and neck squamous cell carcinomas.^9^, ^10^, ^11^, ^12^, ^13^ Recently, a relationship has been shown in many studies between otosclerosis and viral infections.^4^ Although its mechanism has not been fully elucidated, low MPV values have been found in viral diseases.^14^, ^15^, ^16^ Therefore, we hypothesized that low MPV values may be involved in a possible viral etiology which is related to otosclerosis.

To the best of our knowledge, there is no study in the literature that has investigated the relationship between otosclerosis and inflammatory markers that are increasingly used in current ENT practice. The aim of this study was to evaluate the relationship between otosclerosis and markers such as white blood cell (WBC), thrombocytes (Tr), neutrophil lymphocyte ratio (NLR), platelet lymphocyte ratio (TLR), and mean platelet volume (MPV) in patients diagnosed with otosclerosis.

## Methods

A retrospective evaluation was made of the data of patients diagnosed with otosclerosis in the 3 year period of 2015–2018. Patients were excluded if they had liver or kidney failure, a myocardial infarction in the previous 6 months, hyperthyroidism, hypothyroidism, chronic obstructive pulmonary disease, acute or chronic infection, systemic inflammatory rheumatic disease, or if they smoked. A total of 30 otosclerosis patients who met the inclusion criteria and a control group of 30 healthy volunteers with no comorbidities and no smoking history who presented at the otolaryngology clinic were included in the study. The age, gender, BMI and laboratory parameters (WBC, neutrophils, lymphocytes, MPV and Tr) were recorded from the patient files. The NLR was calculated by dividing the neutrophil count by the lymphocyte count and the TLR was calculated by dividing the thrombocyte count by the lymphocyte count. The two groups were compared in respect of WBC, NLR, TLR, MPV and Tr values. Approval for the study was granted by the ethics committee of Mustafa Kemal University. Ethics committee approval's number is 2018/11.

### Statistical analysis

Data obtained in the study were analyzed using SPSS version 21.0 software (IBM, SPSS, Chicago, IL, USA). Continuous data were stated as mean ± Standard Deviation (SD) and categorical data as number (n) and percentage (%). In the comparison of independent groups of continuous data, the Student's *t*-test was used. In comparisons between the groups, the Chi-square test was used in the evaluation of two independent groups of categorical variables. A value of *p* < 0.05 was accepted as statistically significant.

## Results

This retrospective study was conducted on otosclerotic patients who presented at the otolaryngology polyclinic between 2015 and 2018. The otosclerosis patients comprised 12 males and 18 females with a mean age of 39.8 ± 10.0 years and the healthy control group comprised 12 males and 18 females with a mean age of 37.2 ± 8.7 years. No significant difference was determined between the groups with respect to age and gender (*p* = 0.296). When the groups were compared with respect to inflammatory markers, no statistically significant difference was determined for WBC, NR, TLR and Tr values (*p* > 0.05). The MPV values in the otosclerosis group were statistically significantly lower than those of the control group (*p* = 0.047) (Table 1).

**Table 1:** Comparison of clinical and laboratory parameters of both groups.

	Otosclerosis (*n* = 30)	Control (*n* = 30)	*p*-Values
Age, year	39.8 ± 10.0	37.2 ± 8.7	0.296
Sex, (M/F)	12 (40)/18 (60)	12 (40)/18 (60)	1.000
NLO	1.99 ± 0.7	1.72 ± 0.6	0.120
TLO	188.28 ± 231.4	116.40 ± 26.6	0.096
WBC, ×10^3^	7.88 ± 1.3	7.45 ± 2.0	0.335
MPV, fl	9.61 ± 1.4	10.24 ± 1.0	0.047
Platelet, ×10^3^	269.3 ± 83.4	278.2 ± 56.6	0.629

NLO, neutrophil lymphocyte ratio; TLO, platelet lymphocyte ratio; WBC, white blood cell count; MPV, mean platelet volume.

Data are presented as mean ± standard deviation or number (%).

Student's *t*-test or Chi-square test was used.

## Discussion

This is the first study to evaluate the relationship between otosclerosis and inflammatory markers. Although the etiology of otosclerosis is not fully known, various studies have shown that chronic inflammation plays a role in the pathogenesis.^6^ The reason for this inflammatory reaction in the middle ear and sclerotic foci has not been determined, but genetic factors, trauma and vascular, hormonal, autoimmune and viral agents are among the factors thought to be responsible.^3^

The aim of this study was to evaluate the relationship between otosclerosis and the known inflammatory markers of WBC, NLR, TLR, MPV and Tr. The results of the study demonstrated that there was no relationship between otosclerosis and the WBC, NLR, TLR and Tr values, but the MPV values in the otosclerosis patient group were statistically significantly lower than those of the Control Group.

Otosclerosis is a primary disease of the stapes and boney labyrinth, exhibiting an autosomal dominant inheritance, which can cause progressive conductive type and sensoneurial types of hearing loss.^1^ The process of the development of hearing loss shows variability and is usually seen between the third and fifth decades of life.^6^

A series of inflammatory events resulting in bone reorganization triggers the process of bone construction-destruction. In immunohistochemical examinations, the determination in otosclerosis foci of CD3^+^, CD4^+^ and CD8^+^ T lymphocyte cells, complementary fragments and β2 microglobulin, has been evaluated as evidence of the inflammatory mechanism of otosclerosis.^6^ A previous study showing that transforming growth factor (TGF)-β1 has a role in the pathogenesis of various inflammatory diseases demonstrated a relationship with otosclerosis.^6^ In another study, CD8^+^ T lymphocytes and osteoclasts were found around the focus of active otosclerosis and the cytokines expressed were reported to have a role in the etiopathogenesis of otosclerosis.^17^

Neutrophils, lymphocytes and thrombocytes are blood cells that have a function in the inflammatory process. The use of NLR and TLR ratios are markers of systemic inflammation and can be easily calculated and are extremely low cost tests.^18^ There has been research into the relationship between NLR and vestibular neuritis, facial nerve paralysis, sudden sensoneurial hearing loss, squamous cell carcinoma and severe tinnitus, and increased NLR values have been observed in these diseases.^9^, ^10^, ^11^, ^12^, ^13^ Ulu et al. determined that NLR values in patients with sudden hearing loss were statistically significantly higher than those of a control group and the response to treatment was lower in those with high values.^19^ In the current study, no statistically significant difference was determined between the otosclerosis patients and the control group in respect of NLR values (*p* = 0.120).

The thrombocyte-lymphocyte ratio (TLR) is an inflammatory marker showing chronic inflammation similar to NLR. Thrombocytes are necessary for the formation of hemostasis and impairment in this function leads to hemorrhage and coagulation disorders.^20^ In previous studies, TLR values have been found to be high in vascular diseases, coronary artery diseases, chronic kidney failure and gynecological, hepatobiliary and head and neck malignancies,and has been associated with a poor prognosis.^21^, ^22^ When the otosclerosis patients and the control group in the current study were compared in respect of TLR and Tr values, there was no statistically significant difference between the groups (*p* = 0.096, *p* = 0.629).

A relationship has been shown in previous studies between high MPV levels and cerebrovascular events, cardiovascular disease, deep vein thrombosis, diabetes mellitus, and obstructive sleep apnea syndrome. There is thrombocyte activation and aggregation in these diseases.^23^ MPV is a marker showing thrombocyte activation and thrombocytes larger than average have prothrombotic effects. In otolaryngology practice, a relationship has been determined between elevated MPV and chronic nasal obstruction.^24^ However, Karli et al. stated that MPV was not a predictive marker for patients who developed sudden sensorineural hearing loss.^25^ Similarly, Özbay et al. did not determine any relationship between MPV and tinnitus.^12^ Low MPV is related to a reduced number of newly-produced thrombocytes and this reduces the volume and function of thrombocytes. Vascular malformations and inflammatory conditions involving the bowel are often associated with a decrease in MPV. Furthermore, anemia, chronic renal failure, appendicitis, pancreatitis, inflammatory bowel diseases, sepsis, viral infections (Respiratory syncytial virus ? RSV, Epstein–Barr virus, HIV, rubella, etc.) may be associated with a decreased MPV.^14^, ^15^, ^16^ However, the mechanism of low MPV values that are seen in viral infections has not been fully elucidated. They suggested that viruses such as rubella, measles, respiratory syncytial virus play a role in etiopathogenesis of otosclerosis. On the other hand, McKenna and colleagues showed that there is a relationship between measles and otosclerosis in their study. Considering the relationship between otosclerosis and viral infection, our results support the above studies. Low MPV values may suggest the possibility of a viral etiology. In the current study, a statistically significant difference was determined between the groups in respect of the mean MPV values, with the values in the ostosclerosis patient group found to be lower (*p* = 0.047).

There are some limitations to this research. The most significant limitation is being a retrospective study. A second limitation is, that with the exception of the hemogram, there was no evaluation of additional laboratory markers such as C-reactive protein, erythrocyte sedimentation rate, procalcitonin and cytokines, or of hearing test results. Prospective studies are needed to investigate the relationship between low MPV and disease pathogenesis in patients with otosclerosis.

## Conclusion

In conclusion, otosclerosis is a disease with multifactorial etiology, with an active inflammatory process, the etiology of which is not clearly known. However, in the light of the results of this study, the inflammatory markers of NLR, TLR, WBC and Tr should not be considered as predictive markers; however, MPV, may be associated with a possible viral etiology in patients with otosclerosis.

## Conflicts of interest

The authors declare no conflicts of interest.
